# Impact of *Porphyromonas gingivalis* Peptidylarginine Deiminase on Bacterial Biofilm Formation, Epithelial Cell Invasion, and Epithelial Cell Transcriptional Landscape

**DOI:** 10.1038/s41598-018-32603-y

**Published:** 2018-09-20

**Authors:** Ardita Aliko, Marta Kamińska, Brith Bergum, Katarzyna Gawron, Małgorzata Benedyk, Richard J. Lamont, Stanisław Malicki, Nicolas Delaleu, Jan Potempa, Piotr Mydel

**Affiliations:** 1 Broegelmann Research Laboratory, Department of Clinical Science, University of Bergen, N-5021 Bergen, Norway; 2 Department of Microbiology, Faculty of Biochemistry, Biophysics and Biotechnology, Jagiellonian University, 30-387 Kraków, Poland; 3 Małopolska Center of Biotechnology, Jagiellonian University, 30-387 Kraków, Poland; 4 University of Louisville School of Dentistry, Department of Oral Immunology and Infectious Diseases, Louisville, KY 40202 USA; 5 2C SysBioMed, 6646 Contra, Switzerland

## Abstract

Peptidylarginine deiminase (PPAD) is a virulence factor unique to pathogenic *Porphyromonas* species, especially *P. gingivalis*. Mechanistically, PPAD activity, in conjunction with Arg-specific gingipains, generates protein fragments with citrullinated C-termini. Such polypeptides are potential *de novo* epitopes that are key drivers of rheumatoid arthritis. This process could underlie the observed clinical association between rheumatoid arthritis and periodontitis. However, the role of PPAD in host colonization by *P. gingivalis* and, subsequently, in triggering periodontitis is not known. Therefore, the aim of the current study was to delineate the role of PPAD in bacterial biofilm formation, and to define whether adherence to, invasion of, and host responses to bacteria of gingival keratinocytes depend on PPAD activity. We studied these aspects using PPAD-competent and PPAD-incompetent strains of *P. gingivalis*, and demonstrated that neither biofilm formation nor its composition was affected by PPAD activity. Similarly, flow cytometry revealed that PPAD did not impact the ability of *P. gingivalis* to adhere to and, subsequently, invade keratinocytes. Network analyses of gene expression patterns, however, revealed a group of host genes that were sensitive to PPAD activity (*CXCL8*, *IL36G*, *CCL20*, and *IL1B*). These genes can be categorized as potent immune modulators belonging to the interleukin 1 system, or chemoattractants of lymphocytes and neutrophils. Thus, we conclude that PPAD, although it is a potent modulator of the immune response, does not affect bacterial biofilm formation or the ability of *P. gingivalis* to adhere to and invade gingival epithelial cells.

## Introduction

Periodontitis is a chronic inflammatory disease of the tooth-supporting tissues. It is characterized by a progressive loss of attachment caused by the destruction of the connective tissue and alveolar bone, which can lead to tooth loss^1^. Severe-type periodontitis affects about 11% of all individuals globally, while mild to moderate periodontitis affects nearly half of the population^2^. Moreover, periodontitis is associated with various systemic conditions, including atherosclerotic cardiovascular disease^3^, diabetes^4^ and rheumatoid arthritis^5^. The complex pathogenesis of periodontitis implies interplay between the host response, cumulative effects of various risk factors, and bacterial challenge posed by the dental plaque^6,7^.

While over 600 bacterial species have been identified in specimens collected from periodontal pockets, a smaller subset are considered periodontal pathogens. The latter include *Porphyromonas gingivalis*, a Gram-negative anaerobic bacterium, considered to be a major etiologic agent and keystone pathogen of periodontitis^8^. The most prominent and specific virulence factors of *P. gingivalis* are gingipains, cysteine proteinases specific for Arg-Xaa or Lys-Xaa peptide bonds, as well as fimbriae and lipopolysaccharides. In conjunction, they appear to play a crucial role in the development and progression of periodontal disease, either directly or indirectly, by modulating the host inflammatory response^9,10^.

Recently, *P. gingivalis* was shown to be unable to trigger periodontitis in germ-free mice despite its capacity to colonize the host^11^. This underlies the dependency of *P. gingivalis* on accessory or other periodontal pathogens, such as *Tannerella forsythia*, to realize its pathogenic potential. It is well documented that *P. gingivalis* can adhere to many microorganisms that form the dental plaque. These include oral streptococci, *Streptococcus gordonii* in particular, and also *Actinomyces naeslundii*, *Fusobacterium nucleatum*, *T. forsythia*, and *Treponema denticola*^12^. Because *P. gingivalis* is able to induce dysbiosis in microbial communities, it has been postulated that it acts as a keystone pathogen within biofilms^8^. In this context, aggregation of *P. gingivalis* with other bacterial strains present in the oral cavity seems to lead to interactions that are fundamental to inflammation and, ultimately, the development of periodontitis. *P. gingivalis* can also adhere to and invade cells of the gingival epithelium^13^, where it rapidly replicates and accumulates in the perinuclear region^14^. The ability to survive intracellularly allows *P. gingivalis* to evade immune surveillance and antibiotic treatment. It also likely constitutes a critical factor shaping the host cell responses and enabling the progression of periodontitis. A variety of cell-surface and extracellular components, including fimbriae, gingipains, and hemagglutinins, contribute to the adhesive properties of *P. gingivalis*; among these, fimbriae play a key role in attachment to a variety of substrates^15^.

Uniquely among prokaryotes, some pathogenic *Porphyromonas* species, including *P. gingivalis*, produce peptidylarginine deiminase (PPAD)^16^. This enzyme catalyzes the conversion of protein-arginine to protein-citrulline, also referred to as citrullination. In mammals, protein citrullination is a naturally occurring post-translational modification, catalyzed by autologous peptidylarginine deiminase (PADs). PAD enzymes regulate a multitude of physiological processes such as keratinization, myelin sheath stability, and inflammation^17^. In addition to playing an important role in mammalian physiology, protein citrullination is implicated in the pathogenesis of several diseases, including rheumatoid arthritis, certain types of cancer, multiple sclerosis, and Alzheimer’s disease^17^. Highly relevant in this context is the fact that PPAD targets both bacterial and host proteins^18,19^. However, unlike its eukaryotic counterparts, PPAD preferentially citrullinates C-terminal arginines of peptides and can act in a calcium-independent manner^20^. Thus far, most PPAD-related studies have focused on its role in the formation of autoantigens that drive autoimmunity in rheumatoid arthritis. However, no studies have addressed the direct impact of citrullination on bacterial adhesive properties and on the ability of *P. gingivalis* to act as a keystone species driving biofilm formation, or its role in the invasiveness of *P. gingivalis*.

Thus, in the current study, we investigated whether *P. gingivalis* PPAD affects biofilm formation, or adhesion to and invasion of gingival keratinocytes. Since the latter comprise a major interface exploited by colonizing periodontal organisms, we also tested the role for PPAD in shaping host responses.

## Results

### PPAD activity does not affect biofilm formation and its relative composition

**Figure 1:**
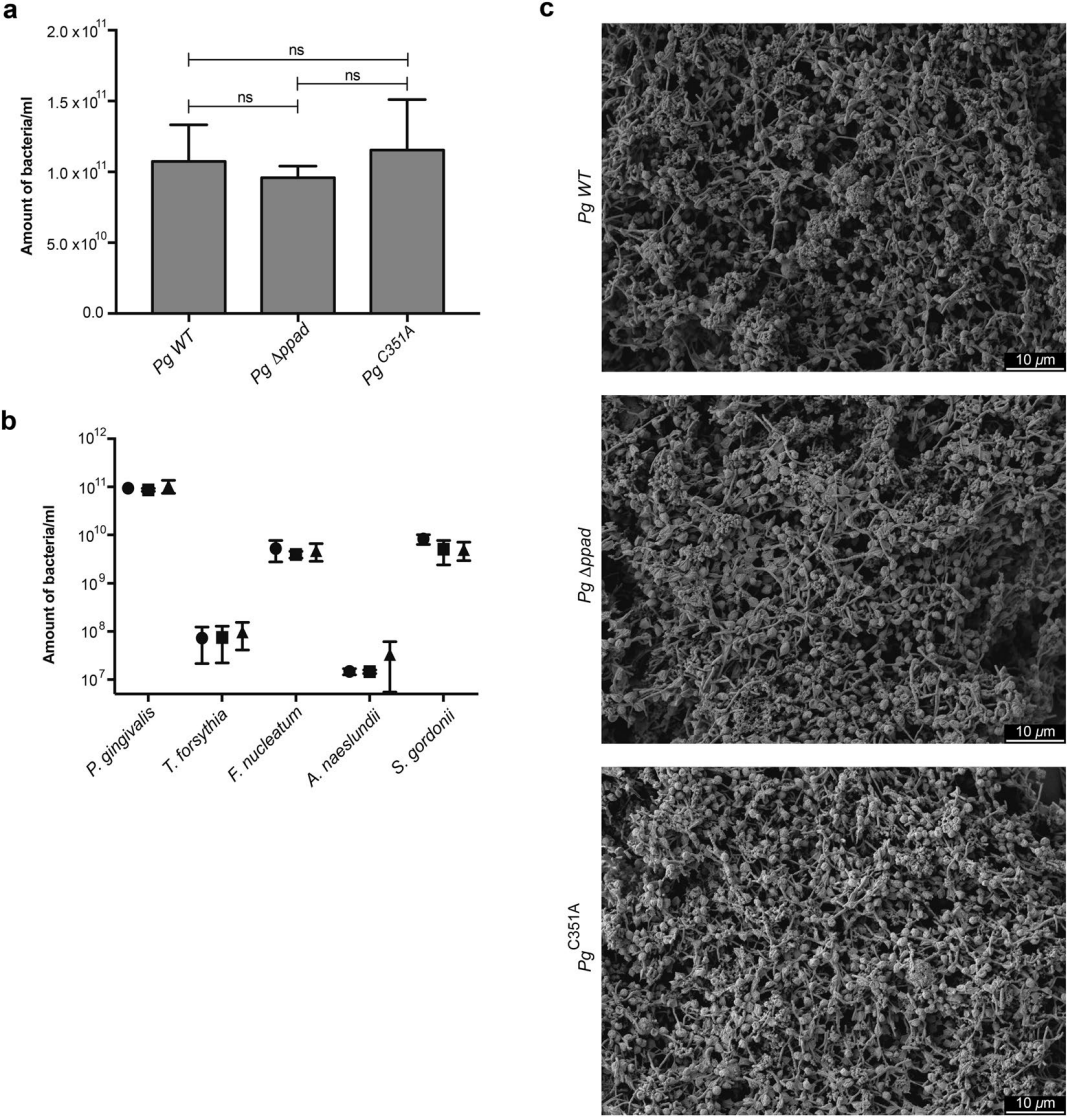
The effect of *P. gingivalis* PPAD on bacterial abundance (**a**), species composition (**b**) and (**c**) the biofilm structure in multispecies biofilms. A biofilm consisting of *T. forsythia*, *F. nucleatum*, *A. naeslundii*, *S. gordonii*, and one of three *P. gingivalis* strains, wild type (WT), *ppad* deletion strain (*Δppad*), or a strain harboring inactivated PPAD (C351A), was cultured for 48 h, following which (**a,b**) the bacterial DNA was extracted and assessed via qPCR, (**c**) biofilm was fixed and observed via SEM under 1500x magnification. The (**a**) bacterial load and (**b**) species composition of each biofilm model are presented as mean ± SD from three independent experiments. Data were plotted on a logarithmic scale.

To investigate the role of protein citrullination in biofilm formation, a five-species biofilm model mimicking the subgingival plaque was used. To assess the direct contribution of PPAD in this model, the abundances of the individual species within the different biofilms were quantified by qPCR. Interestingly, the lack of PPAD activity did not affect the qualitative and quantitative composition of the multispecies biofilm (Fig. 1). Furthermore, PPAD had no impact on the biofilm structure (Fig. 1) or the viability of cells (94,13 ± 0,39% viability on average for the biofilm with wild type (WT) *P. gingivalis*, 93,49 ± 0,71% for the biofilm with *P. gingivalis* Δppad, and 93,07 ± 1,21% for the biofilm with *P. gingivalis* C351A).

### PPAD activity does not impact the capacity of ***P. gingivalis*** to adhere to and be internalized by TIGKs

**Figure 2:**
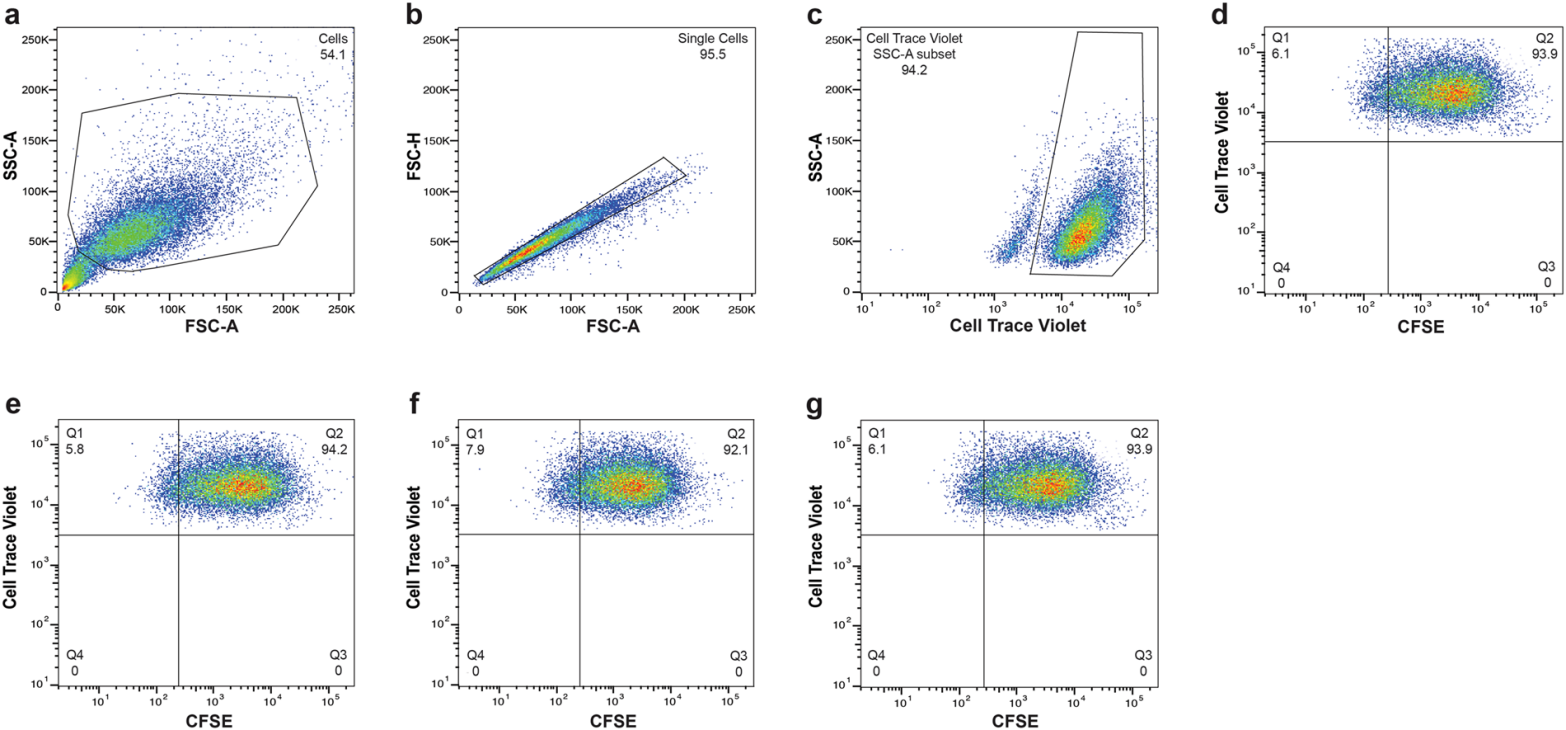
*P. gingivalis* invasion of human oral keratinocytes. CTV-labeled TIGKs were infected with various CFSE-labeled strains of *P. gingivalis* (MOI = 200) for 90 min. Metronidazole was added for an additional 1 h, following which the cells were fixed and analyzed by flow cytometry. Cells were first gated on the basis of a forward scatter/side scatter (FSC-A/SSC-A) plot (**a**). The events were then visualized using FSC-A/FSC-H dot plot, and single cells were gated (**b**). TIGKs were identified on the basis of CTV positivity (**c**). TIGKs invaded by *P. gingivalis* were subsequently defined as the CFSE^+^ cells within the CTV^+^ keratinocyte population (**d**). Overall, 20,000 events were analyzed, and the results are presented as the percentage of TIGKs infected with *P. gingivalis* wild-type (**e**), *Δppad* (**f**), and C351A (**g**).

The ability to adhere to and invade epithelial cells is one of the most important features that allow bacteria to cross the mucosal barrier and infect tissues. Thus, we examined whether PPAD-dependent citrullination of the bacterial or host proteins played a role in these processes. We used CTV-stained keratinocytes and CFSE-stained *P. gingivalis* cells to evaluate cell attachment and invasion. In contrast with previous reports using primary human gingival fibroblasts^21^, both PPAD mutants adhered to and invaded keratinocytes to the same extent as the parental strain. After 90 min of incubation at MOI = 200, the two PPAD mutants adhered to and invaded TIGKs as efficiently as *P. gingivalis* WT. Under each of the tested conditions, more than 90% of TIGKs were attached to or internalized *P. gingivalis*, irrespective of the PPAD activity (WT: 95.9 ± 3.4%; Δ*ppad*: 96.4 ± 4.1%; and C351A: 96.7 ± 3.8%). The samples were treated with metronidazole to remove any non-internalized bacteria from the TIGK surface, and the subsequent flow cytometry further confirmed the lack of relatedness between the extent of internalization and PPAD activity. Regardless of the *P. gingivalis* strain used, the bacteria infected more than 90% of TIGKs (WT: 94.7 ± 3.5%; Δ*ppad*: 94.2 ± 4.3%; and C351A: 95.8 ± 2.3%) (Fig. 2).

### The effect of PPAD on TIGK gene expression

**Figure 3:**
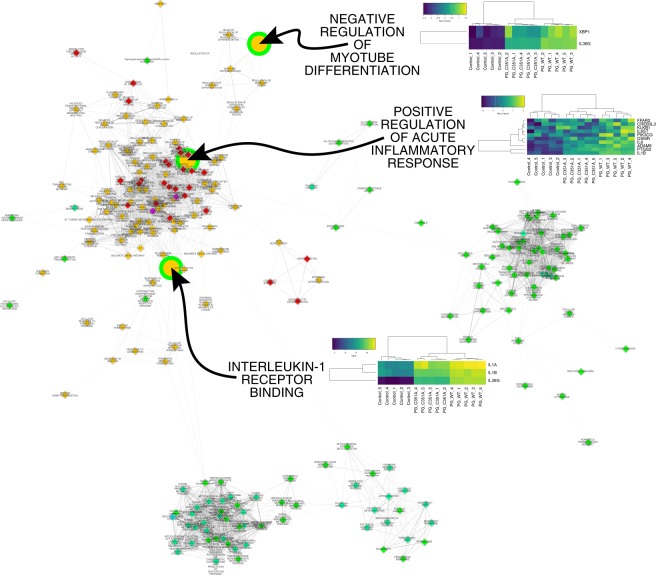
Alteration of the transcriptional landscape of TIGKs infected with *P. gingivalis* in the presence of absence of active PPAD. Significant changes in GS, *e.g*., biological processes and pathways, induced in TIGKs upon infection with PPAD-competent *P. gingivalis* were mapped. The fill color of each node represents the direction and significance level for this comparison (orange, enrichment, FDR q < 0.05; red, enrichment, FDR q < 0.01; magenta, enrichment, FDR q < 0.001; green, depletion, FDR q < 0.05; spring green, depletion, FDR q < 0.01; cyan, depletion, FDR q < 0.001). Alterations observed upon comparing TIGKs infected with *P. gingivalis* PPAD^C351A^ (inactive PPAD) and TIGKs infected with *P. gingivalis* WT are superimposed on this network. The result of the comparison is visualized by the border color of each node (green, depletion, FDR q < 0.05; gray, unchanged). The alterations significantly dependent on PPAD activity were magnified. For each of these pathways, heatmaps of the genes significantly contributing to their enrichment in TIGKs infected with *P. gingivalis* WT vs. uninfected cells, and significantly contributing to their depletion in TIGKs infected with *P. gingivalis* PPAD^C351A^ vs. TIGKs exposed to *P. gingivalis* WT, are given. Each field of the heat map is color coded to represent the per-row z-score of the gene expression value of the respective gene and sample.

The data thus far indicated that the adherence to and internalization of *P. gingivalis* by TIGKs are independent of the PPAD activity. To extend these analyses and delineate in an unbiased fashion the potential effects of PPAD inactivation on TIGKs, TIGK gene expression profiles were evaluated. Out of 10,219 analyzed GSs (*i.e*., *a priori* defined groups of genes, compiled, curated, and annotated to reflect one specific trait shared by their members, *e.g*., keratinocyte differentiation), 239 processes were significantly altered when TIGKs infected with *P. gingivalis* were compared with the uninfected controls (Fig. 3). Among these pathways, only three, *(i)* interleukin-1 (IL-1) receptor binding (GO:0005149), *(ii)* positive regulation of acute inflammatory response (GO:0002675), and *(iii)* negative regulation of myotube differentiation (GO:0010832) appeared to be dependent on PPAD as they were significantly depleted when TIGKs infected with *P. gingivalis* C351A were compared with TIGKs infected with *P. gingivalis* WT. Underlying the modulation of these three processes were a total of 13 genes (leading-edge genes). They contributed to *(i)* the significant enrichment in *P. gingivalis* WT versus uninfected controls and also *(ii)* to the significant depletion of the same respective pathway when comparing TIGKs infected with *P. gingivalis* C351A versus TIGKs infected with *P. gingivalis* WT (Fig. 3). Among these 13 genes we noticed a predominance of IL-1 and IL-6 system-related genes.

**Figure 4:**
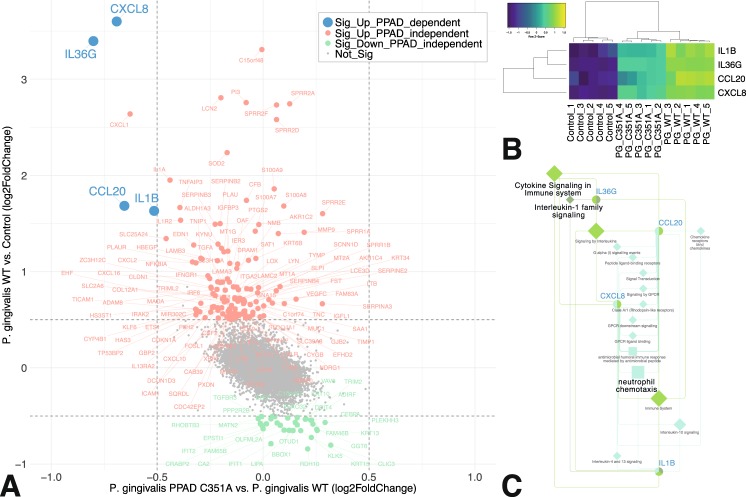
Identification and functional annotation of TIGK genes significantly dependent on PPAD activity. (**A**) Scatterplot of log2 fold-change (log2FC) in gene expression of TIGKs infected with *P. gingivalis* WT vs. control (y-axis), and *P. gingivalis* PPAD^C351A^ vs. *P. gingivalis* WT (x-axis). Genes altered exclusively in the *P. gingivalis* WT vs. control comparison are displayed in red when significantly up-regulated (log2FC > 0.5, FDR q < 0.05) and in green when significantly down-regulated (log2FC > 0.5, FDR q < 0.05). Genes showing a significant degree of dependence on PPAD activity (significant up-regulation in the *P. gingivalis* WT vs. control comparison, and significant down-regulation in the *P. gingivalis* PPAD^C351A^ vs. *P. gingivalis* WT comparison) are highlighted in blue. (**B**) Clustered heatmap of PPAD-dependent genes. (**C**) Functional annotation of PPAD-dependent genes in the GS: gene network. The latter connects PPAD-dependent genes (blue font) with key terms (black font) and the associated terms (gray font) identified using ClueGO. Each field of the heat map is color coded to represent the per-row z-score of the gene expression value of the respective gene and sample.

Focusing on differential expression of single genes instead of gene patterns, the expression of *CXCL8*, *IL36G*, *CCL20*, and *IL1B* was significantly PPAD-dependent (Fig. 4A,B). Again, functional annotation highlighted immune system signaling and triggering of the immune cell chemotaxis. This confirmed the findings of the network analyses delineated in Fig. 3 and reinforced the notion that host genes that are sensitive to PPAD in this system belong to specific classes of immune modulators from the IL-1 system and chemoattractants signaling lymphocytes, and neutrophils.

## Discussion

Citrullination is an enzymatic post-translational modification that converts the positively charged side chain of arginine into a neutral citrulline residue. Citrullination may dramatically modify the structure of peptides and proteins, abrogating or changing their function^17^. The pathophysiological role of human PADs was studied intensely in the past several years. More recently, the unique microbial PAD emerged as a relevant player in various inflammatory diseases. It was initially thought that the enzyme was produced exclusively by *P. gingivalis* but, recently, putative animal pathogens from the *Porphyromonas* family, including *P. gulae* and *P. loveana*, were shown to produce it as well^16^. The presence of a nearly identical enzyme in related pathogens further underlines its importance as a virulence factor. In contrast to human PADs, PPAD acts in a calcium-independent manner and nearly exclusively citrullinates carboxyterminal arginine residues^20^. In *P. gingivalis*, PPAD acts in concert with Arg-gingipains, which generate protein fragments and peptides with carboxyterminal arginine residues that are subsequently citrullinated by PPAD^18^. Thus far, it has been shown that PPAD-induced citrullination affects complement activity^22^, inactivates epidermal growth factors^23^, and may contribute to the alveolar bone loss by activating the prostaglandin E2 signaling pathway in fibroblasts^21^. Moreover, this reaction can generate citrullinated epitopes that are important in the pathogenesis of autoimmunity in general and rheumatoid arthritis in particular^18,24^.

It is conceivable that citrullination of *P. gingivalis* surface proteins involved in the adhesion and invasion of host cells^15^ would also determine its adherence to other bacteria and to host cells. Gawron *et al*. showed that PPAD activity enhances bacterial adherence and invasion of fibroblasts^21^. This effect was not observed during *P. gingivalis* interaction with keratinocytes in the current study. Nonetheless, it is possible that the difference in cell types used in our study and in the study by Gawron *et al*.^21^ accounts for this lack of accordance. First, in contrast to Gawron *et al*., where primary human gingival fibroblasts were used, our study employed an epithelial cell line. Primary cells are believed to be more biologically relevant tools than cell lines; however the TIGK cell line used in our study was shown to have similar characteristics to the parental cell type^25^. Importantly, TIGKs were also comparable to primary gingival epithelial cells in levels and kinetics of invasion by wild type *P. gingivalis* and an invasion defective Δ*serB* mutant^25^. Moreover, keratinocytes and fibroblasts are different cell types. Keratinocytes reside in the outermost layer of the epithelium, representing the first cell barrier encountered by bacteria. Fibroblasts, on the other hand, typically reside deep within the connective tissue. These observations may therefore indicate that cell type-specific differences in pathogen adherence and internalization occur in relation to citrullination. Indeed, Sipilä *et al*. demonstrated that citrullination of collagen II significantly affected its binding by integrins α_10_β_1_ and α_11_β_1_^26^. These integrins are expressed on fibroblasts and chondrocytes, but not on keratinocytes^26^, and one may speculate that collagen citrullination specifically affects the function of these cell types. Concomitantly, citrullination of collagen does not affect its interaction with the α_1_β_1_ and α_1_β_2_ receptors, suggesting that the impact of collagen citrullination on the function of inflammatory cells predominantly expressing α_1_β_1_ and α_1_β_2_ integrins is not as pronounced. Of note, adhesion of *P. gingivalis* FimA fimbriae to the epithelial cells of the gingiva is mediated by the α_5_β_1_ integrin^12,27^, whereas fimbrial adhesion to other bacteria depends on various receptors, *e.g*., GAPDH and SspA/B surface proteins of *S. gordonii*^28,29^. Based on our findings, it seems unlikely that citrullination by *P. gingivalis* PPAD affects those interactions.

Although the PPAD-encoding gene is strictly conserved in laboratory strains and clinical isolates of *P. gingivalis*^19^, the citrullination capacity of different strains has only recently been tested, in two studies^18,30^. Wegner *et al*. showed that endogenous protein citrullination is pronounced in *P. gingivalis* W83 and four clinical isolates from patients with periodontal disease^18^. Furthermore, sub-cellular fractionation revealed that the majority of citrullinated proteins were associated with the periplasm and the cell envelope fraction. Obviously, the latter are critical for *P. gingivalis* adherence and interaction with other cells. A recent mass spectrometry-based study uncovered a substantial strain-dependent heterogeneity of the extracellular proteome and citrullinome of *P. gingivalis*^30^. Interestingly, the extracellular citrullinome consisted of only a few proteins, including, in almost all strains, an arginine-specific gingipain. In addition, four proteins with unknown function, mainly present in clinical isolates, and a minor fimbrial protein (Mfa1) were citrullinated in some isolates, but not in the ATCC 33277 reference strain. Importantly, no citrullination of the major fimbrial protein FimA was detected; FimA is regarded as the key *P. gingivalis* protein responsible for its adhesion properties^30^.

We also investigated the impact of PPAD on the host cell cytokine responses to *P. gingivalis*. PPAD was especially important for the expression of IL-36γ in human gingival epithelial cells, although it was also associated with increased expression of IL-8, IL-1β, *CCL20*, and *CXCL8*. IL-36 is a collective name for three novel members of the IL-1 proinflammatory cytokine family (IL-36α, β, and γ) that are all highly expressed in epithelial tissues and in several myeloid-derived cell types^31^. Growing evidence supports the role of IL-36 as a potent regulator of dendritic and T-cell responses^31^, and its importance in inflammatory disorders. Currently, little is known about the function of IL-36 in the pathogenesis of periodontitis. Notably, unlike IL-36α or IL-36β, IL-36γ was only recently identified as one of the most strongly induced inflammatory genes of the human oral epithelial cells following *P. gingivalis* infection^32^. These findings are in agreement with those of the current study, and consistent with the notion that IL-36γ plays an important role in the host response to *P. gingivalis*. Furthermore, IL-36γ stimulates the expression of the neutrophil chemokine IL-8, as well as the Th17 chemokine CCL20^32^. Indeed, Th17 cells represent the predominant T-cell subset in the chronic periodontitis lesions^33^. Therefore, IL-36γ might act as an important mediator of the *P. gingivalis*-driven Th17 polarization, with PPAD likely contributing to its expression. Overall, network analysis performed in the current study reinforces the notion that host genes that are sensitive to PPAD activity belong to specific classes of immune modulators from the IL-1 system and chemoattractants signaling lymphocytes, and especially neutrophils. This suggests that PPAD is an important immune regulator with a role in the pathogenesis of periodontitis. In agreement with this hypothesis, a recent study in a murine model for experimental periodontitis has demonstrated that oral inoculation with the PPAD-deficient strain resulted in a significantly reduced amount of periodontal bone loss compared to oral inoculation with the wild type *P*. gingivalis^34^.

In conclusion, the current study demonstrated that PPAD activity is not essential for the incorporation of *P. gingivalis* in multispecies biofilms and does not affect composition in these biofilms. Furthermore, PPAD activity apparently did not contribute to the ability of *P. gingivalis* to adhere to and infect human oral keratinocytes *in vitro*. Nevertheless, in host cells infected with PPAD-incompetent strains, the expression of genes related to cytokine production was significantly reduced in comparison with that in cells infected with *P. gingivalis* WT. This observation indicates that PPAD may exert an important effect on the immune landscape of periodontitis, which should be investigated further in more detail.

## Materials and Methods

### Bacterial strains and mammalian cell culture

Wild-type (WT) *P. gingivalis* strain ATCC 33277, its isogenic mutants with a deleted PPAD gene (Δ*ppad*) and expressing catalytically inactive PPAD (C351A, PPAD^C351A^), and *T. forsythia* ATCC 43037, *F. nucleatum* ATCC 25586, *A. naeslundii* ATCC 12104, and *S. gordonii* ATCC 10558 were used in this study. *T. forsythia* was grown on ATCC 1921-NAM agar, and the remaining strains were pre-cultivated on blood agar. The bacteria were maintained at 37 °C anaerobically (under 85% N_2_, 10% H_2_, and 5% CO_2_), except for *S. gordonii*, which was grown aerobically. The biofilm growth medium was the modified Wilkins-Chalgren anaerobe broth supplemented with 5% defibrinated sheep blood and 0.01% *N*-acetylmuramic-acid (Sigma-Aldrich, St. Louis, USA). Telomerase-immortalized gingival keratinocytes (TIGKs)^25^ were cultured in the keratinocyte growth medium, supplemented with hydrocortisone, epidermal growth factor, insulin, epinephrine, transferrin, and bovine pituitary extract (KGM Gold; Lonza, Basel, Switzerland) at 37 °C under 5% CO_2_. Keratinocytes were used at passages 3–5 at a confluence of 80%.

### Multispecies biofilm formation

A biofilm model consisting of five bacterial species was used. *P. gingivalis* (WT or its isogenic mutants) and the remaining four species: *T. forsythia*, *F. nucleatum*, *A. naeslundii*, and *S. gordonii*. The optical density of bacterial cultures was adjusted to OD_600_ = 1.0 ± 0.05, and the cultures were diluted 1:12.5 in the biofilm growth medium. The fast-growing *S. gordonii* was further diluted 1:10. Per well, 125 μl each of the *P. gingivalis*, *F. nucleatum*, and *T. forsythia* suspensions, and 65 μl each of the *A. naeslundii* and *S. gordonii* suspensions, were added, and mixed, for a final total volume of 500 μl, which was then transferred to 24-well plates pre-coated with 0.01% poly-l-lysine (Sigma-Aldrich). The bacteria were incubated anaerobically at 37 °C for 48 h. The planktonic cells were removed and the biofilm layer was harvested. Bacterial composition in the resulting suspensions was determined by a quantitative real-time polymerase chain reaction (qPCR).

### Quantitative real-time PCR (qPCR)

DNA was extracted using the Chelex™ extraction technique. Briefly, samples were centrifuged, the supernatant removed, and 200 μl of 6% Chelex^*®*^ 100 resin (BioRad, Hercules, USA) solution was added to the pellets. The samples were then incubated at 56 °C for 30 min, vortex-mixed at high speed for 10 s, incubated at 100 °C for 8 min, vortex-mixed again at high speed for 10 s, and spun down for 2 min. The DNA content in the supernatant was determined using NanoDrop ND-1000 (ThermoFisher, Wohlen, Switzerland). Samples were stored at −20 °C.

To quantify the relative proportions of each species in the biofilms, species-specific primer pairs were used: *P. gingivalis* forward, 5′-AGGCAGCTTGCCATACTGCG-3′, and reverse, 5′-ACTGTTAGCAACTACCGATGT-3′; *S. gordonii* forward, 5′-GCACTTGCAAAACACCCTGAA-3′, and reverse, 5′-ACGAGTTGTTGCTGCAGTTG-3′; *F. nucleatum* forward, 5′-AGAGTTTGATCCTGGCTCAG-3′, and reverse, 5′-GTCATCGTGCACACAGAATTGCTG-3′; *T. forsythia* forward, 5′-GCGTATGTAACCTGCCCGCA-3′, and reverse, 5′-TGCTTCAGTGTCAGTTATACCT-3′; and *A. naeslundii* forward, 5′-CTCCTACGGGAGGCAGCAG-3′, and reverse, 5′-CACCCACAAACGAGGCAG-3′.

The qPCR was performed in a total reaction volume of 50 μl, containing 25 μl of GoTaq® qPCR MasterMix (Promega, Madison, USA), 10 μl of the extracted DNA, and the species-specific primer pair (final concentration of each primer: 0.2 μM). Amplification of the extracted DNA template was performed using the LightCycler 480 instrument (Roche, Pleasanton, USA), with an initial incubation of 2 min at 95 °C; followed by 40 cycles of 15 s at 95 °C and 1 min at 60 °C. The obtained Cq values were used to calculate the amount of bacteria in the sample, with reference to the DNA standards generated for each strain. Melting-curve profiles were examined to verify primer specificity and evaluate the quality of the reaction products.

### Biofilm viability

A 96-well black with clear flat bottom polystyrene microplate (Corning, NY, USA), pre-coated with 0.01% poly-l-lysine, was inoculated with 200 μl/well of the bacterial suspension prepared as described above. After culturing at 37 °C for 48 h in anaerobic conditions, biofilms were washed once with water and stained for 20 min with 100 μl of LIVE/DEAD BacLight Bacterial Viability Kit (Invitrogen, NY, USA). The final concentrations of SYTO 9 and propidium iodide (PI) were 10 μM and 60 μM, respectively. The stained biofilms were observed with a Cytation™ 5 Cell Imaging Multimode Reader (BioTek, Winooski, USA) at excitation wavelengths of 488 nm (SYTO 9) and 568 nm (PI).

### Scanning electron microscopy

The biofilm structure was assessed by scanning electron microscopy (SEM). Biofilm was cultured for 48 h as described above on 12 mm glass slides, then washed with PBS and fixed using 2%  glutaraldehyde in 0.1 M sodium cacodylate buffer, pH 7.4, for 2 h at room temperature, and dehydrated in ascending concentrations of ethanol. Following critical point drying, slides were sputter coated with gold and mounted on stubs. Imaging was performed using Jeol JSM-7400F scanning electron microscope under magnification of 1500X.

### Adherence and invasion assay

TIGKs and *P. gingivalis* ATCC 33277 strains (WT, Δ*ppad*, and C351A) were stained using the CellTrace™ Violet cell proliferation kit (Life Technologies, Paisley, UK) (CTV) and CellTrace™ carboxyfluorescein succinimidyl ester (CFSE) cell proliferation kit (Life Technologies), respectively, according to the manufacturer’s protocols. Labeled keratinocytes were seeded onto 6-well plates. The confluent cell monolayer was infected for 90 min with each strain of the labeled bacteria at a multiplicity of infection (MOI) of 200, and maintained at 37 °C under 5% CO_2_. Unattached extracellular bacteria were removed by PBS washes, and the cells were trypsinized and fixed in 4% paraformaldehyde in PBS for 15 min at room temperature. The cells were washed, re-suspended in PBS, and analyzed using the LSRFortessa™ cell analyzer (BD Biosciences, Franklin Lakes, USA). The cells were gated on forward and side scatter to eliminate debris. Next, they were gated on the following populations: CTV^+^ CFSE^+^, representing TIGKs with bacteria either attached or internalized; CTV^+^ CFSE^−^, representing TIGKs with no bacteria attached or internalized.

A standard antibiotic protection assay was performed to differentiate between the attached and internalized bacteria. After infecting the cell monolayer, an additional step was included: incubating the cells with metronidazole (100 μg/ml; Sigma-Aldrich) for 1 h at 37 °C under 5% CO_2_, to kill the adherent bacteria. TIGK invasion by *P. gingivalis* was then determined by flow cytometry as described above.

### Keratinocyte infection

TIGKs seeded in a 24-well plate were infected with *P. gingivalis* and its PPAD-deficient strains at MOI = 100 for 12 h, in DMEM (ThermoFisher) with 4% FBS. The medium was removed, fresh DMEM containing 4% FBS and gentamicin (ThermoFisher) was added, and the cells were incubated for another for 2 h. Subsequently, the cells were washed thoroughly with PBS and lysed with GeneJET RNA purification kit (ThermoFisher) prior to RNA isolation, performed according to the manufacturer’s instructions.

### RNA-Seq and mapping of the TIGK transcriptional landscape in response to PPAD activity

Global gene expression profiles were generated from the following samples using HumanHT-12 v4 Expression BeadChip kits (Illumina, San Diego, USA): *(i)* uninfected TIGKs; *(ii)* TIGKs infected with *P. gingivalis* WT (ATCC 33277); and *(iii)* TIGKS infected with *P. gingivalis* PPAD^C351A^. Five biological replicates were analyzed per group. The transcriptional landscapes and their visualization in gene set (GS): GS networks were computed as described previously^35^, with slight modifications: *(i)* the GS collection was updated to February 24, 2015 and included the complete transcription factor target and miR target collections from MSigdb (of the 22,710 GS comprised in this collection, 10,219 passed the GS size filter criteria of >10 and <500); *(ii)* to allow the integration of the following two comparisons in the same network, *(1)* TIGKs infected with WT *P. gingivalis* vs. uninfected TIGKs, and *(2)* TIGKs infected with *P. gingivalis* PPAD^C351A^ vs. *P. gingivalis* WT, the pair-wise edge connectivity parameter was not calculated for the shared leading genes but included all genes in the respective GS pairs. In this context, the edge connectivity threshold for a “true” edge was set at >0.05. To preserve network integrity, disconnected nodes were avoided by retaining the edge with the highest connectivity measure <0.05; *(iii)* the significance threshold for a mapped GS was set to a false discovery rate (FDR) q < 0.05; *(iv)* the complexity of the resulting network did not mandate MCL clustering applied in the previous study^35^.

### Differential single-gene expression analyses and functional annotation

Differential gene expression was computed on the same basis as the analyses described above, with the thresholds set to reach significance at FDR q < 0.05; and log2 fold-change set at >0.5 for up-regulation, and at −0.5> for down-regulation. Four genes whose significant up-regulation was deemed to be PPAD-dependent were functionally annotated using ClueGO 2.5.0^36^ within the Cytoscape 3.6.0 suite^37^. The analysis settings were as follows: *(i)* GO hierarchy levels: 3–8; *(ii)* hits required to map a GO term: ≥1; and *(iii)* GO term fusion and grouping applied based on the kappa score threshold of 0.4 (the kappa score is the similarity measure for GO terms). For each group of terms, the GO term accommodating the largest number of MAP proteins was selected as its leading term. GO immune system processes (11.20.2017) as well as reactome pathways were queried (11.20.2017). The same approach to functional annotation is presented in more detail in the work of Delaleu *et al*.^38^ and Delaleu *et al*.^39^.

### Statistical analysis

Each experiment was performed three times, at least in duplicate. Comparisons of the bacterial abundance, composition and total biomass of multispecies biofilm were performed using the nonparametric Kruskal-Wallis tests (SPSS Statistics v.24, IBM, Chicago, USA) with p < 0.05 as a significance threshold.

## Data Availability

The datasets generated during and/or analyzed during the current study are available from the corresponding authors upon request.
